# Psychotropic prescribing for English care home residents with dementia compared with national guidance: findings from the MARQUE national longitudinal study

**DOI:** 10.1192/bjo.2021.21

**Published:** 2021-09-15

**Authors:** Francesca La Frenais, Victoria Vickerstaff, Claudia Cooper, Gill Livingston, Patrick Stone, Elizabeth L. Sampson

**Affiliations:** Division of Psychiatry, University College London, UK, and Marie Curie Palliative Care Research Department, Division of Psychiatry, University College London, UK; Marie Curie Palliative Care Research Department, Division of Psychiatry, University College London, UK; Division of Psychiatry, University College London, UK, and Camden and Islington NHS Foundation Trust, St Pancras Hospital, London, UK; Division of Psychiatry, University College London, UK, and Camden and Islington NHS Foundation Trust, St Pancras Hospital, London, UK; Marie Curie Palliative Care Research Department, Division of Psychiatry, University College London, UK; Marie Curie Palliative Care Research Department, Division of Psychiatry, University College London, UK, and Barnet Enfield and Haringey Mental Health Trust Liaison Team, North Middlesex University Hospital, London, UK.

**Keywords:** Dementia, antianxiety drugs, antipsychotics, antidepressants, carers

## Abstract

**Background:**

Despite policy pressure and concerns regarding the use of antipsychotics and benzodiazepines, many care home residents with dementia are prescribed psychotropic medication, often off licence. This is the first large study to report psychotropic prescribing and ‘as required’ administration patterns in English care homes.

**Aims:**

To explore the prevalence and associates of psychotropic prescription in care home residents with dementia and compare the results with national guidance.

**Method:**

We collected data in a longitudinal cohort study of residents with diagnosed or probable dementia in 86 care homes in England in 2014–2016. We reported the prevalence of psychotropic (antipsychotics, anxiolytics/hypnotics, antidepressants) prescriptions and drug receipt. We explored the associations between resident factors (sociodemographic, agitation [Cohen–Mansfield Agitation Inventory], dementia severity [Clinical Dementia Rating]) and care home factors (type, ownership, size, dementia registration/specialism, quality rating) in prescription and ‘as required’ administration, using multilevel regression models.

**Results:**

We analysed data from 1425 residents. At baseline, 822 residents (57.7%, 95% CI: 55.1–60.2) were prescribed a psychotropic drug, 310 residents (21.8% 95% CI: 19.7–24.0) were prescribed an anxiolytic/hypnotic, 232 (94.3%, 95% CI: 90.6–96.6) were prescribed one antipsychotic and 14 (5.7%, 95% CI: 3.4–9.4) were prescribed two antipsychotics. The median prescription duration during the study was 1 year. Residents with clinically significant agitation were prescribed more antipsychotics (odds ratio [OR] = 2.00, 95% CI: 1.64–2.45) and anxiolytics/hypnotics (OR = 2.81, 95% CI: 2.31–3.40).

**Conclusions:**

Antipsychotics and anxiolytics/hypnotics are more commonly prescribed for people with dementia in care homes than in the community, and prescribing may not reflect guidelines. Policies which advocate reduced use of psychotropics should better support psychosocial interventions.

Care home residents living with dementia frequently have behavioural and psychological symptoms of dementia (BPSD), including restlessness, sleeping problems, and depression. Guidelines recommend that initially non-pharmacological treatments should be used;^1^ psychotropic drugs, primarily antipsychotics, hypnotics, anxiolytics including benzodiazepines, and antidepressants, are often prescribed to treat these symptoms.^2,3^ UK care homes do not typically record medication use electronically; thus, it is difficult to ascertain prescribing prevalence and drug receipt in this population. A study of a large longitudinal cohort in the UK found that, for people with dementia, antipsychotic drug prescriptions decreased from 22.1% in 2005 to 11.4% by 2015, hypnotic prescriptions decreased from 14.3% to 9.5% and antidepressant prescriptions increased from 28.0% to 36.6%.^3^

Guidance published by the Alzheimer's Society recommends that only residents with agitation that causes extreme distress or risk, or those with severe depression, should be prescribed pharmacological treatment, and evidence supporting the effectiveness of these treatments is very limited.^4^ In the UK, all drugs are prescribed on the NHS according to a standard formulary,^5^ and specific dementia guidance has been developed for prescribers. National Institute for Health and Care Excellence (NICE) guidelines state that antipsychotics can be prescribed for patients who are experiencing BPSD that are causing them severe distress, or who are at risk of harming themselves or others, but prescriptions should be regularly reviewed and used for the shortest possible duration.^1,6^ Owing to associated side-effects including sedation, dizziness, increased risk of falls, and cardiac effects, it is advised that these drugs are prescribed initially only for up to 6 weeks until review for BPSD, although an analysis of UK national data (2014–2016) indicated that median durations of antipsychotic prescribing were several times this.^4,6–8^ Care home residents with dementia are prescribed more psychotropics than residents without dementia and people with dementia overall.^3,9^ In the past decade, there has been increased policy pressure, such as in the National Dementia Strategy, to reduce psychotropic use in people with dementia, with particular focus on antipsychotics.^10^ However, practice in care homes where people may have more BPSD may not reflect these policy drivers.^11^ Further, there are concerns that other medications with potentially harmful side-effects, such as benzodiazepines, antidepressants or mood stabilisers, may now be prescribed in place of antipsychotics; these are drugs that are currently not licensed to treat BPSD.

The aim of this study was to explore psychotropic medication use in English care homes. The objectives were as follows.

- To describe the prescription prevalence of antipsychotic, antidepressant, and anxiolytic and hypnotic medication.
- To describe how often as required (‘PRN’) drugs are administered over a 2 week period.
- To explore resident and care home factors associated with psychotropic use.

## Method

### Study design

This study^1^ is part of a longitudinal observational cohort study embedded in the MARQUE (Managing Agitation and Raising QUality of LifE in dementia) programme.^12^ The authors assert that all procedures contributing to this work comply with the ethical standards of the relevant national and institutional committees on human experimentation and with the Helsinki Declaration of 1975, as revised in 2008. All procedures involving human subjects/patients were approved by the London (Harrow) National Research Ethics Service Committee (14/LO/0034).

### Setting

We collected data in English care homes (nursing care and residential care). Care homes were eligible for recruitment if they had residents with dementia and were selected to be representative of provider type (private, third sector or state) and care provision (nursing or residential). Study recruitment and data collection took place between May 2014 and December 2016.

### Participants

Residents were deemed eligible if they were diagnosed with dementia by a clinician or if they scored two or above on the Noticeable Problems Checklist (NPC).^13^ The NPC is validated against clinical diagnoses of dementia.^14^ Eligible residents were invited to take part; initially the care home approached the resident or their next of kin (if they deemed it likely that the resident would lack capacity to consent). If the resident or next of kin agreed that the research team could contact them, then we sought written or verbal informed consent or consultee agreement. If there was no next of kin then we asked a familiar care home staff member to act as a professional consultee. We witnessed and formally recorded verbal consent.

### Data sources and measurement

Care home and resident demographics were recorded at baseline. For care homes these characteristics were: size (number of beds), care provision, dementia registration, dementia specialism and Care Quality Commission rating. For residents the characteristics were: age, sex, ethnicity, first language (either English or other) and whether they had a dementia diagnosis. At each study visit (baseline, 4 months and 12 months), a researcher conducted interviews with care staff who were familiar with the participants, using validated instruments to assess agitation (Cohen–Mansfield Agitation Inventory; CMAI^15^) and severity of dementia (Clinical Dementia Rating; CDR^16^). Medication data were collected at each study visit from Medication Administration Records (used in all recruited care homes): drug, dosage, frequency and whether the prescription was regular or PRN; if PRN, the number of times it was administered in the previous 2 weeks (to coincide with the CMAI data) and the indications were recorded.

### Study size

The sample size calculation for MARQUE used data from a prior research study, START.^17^ In the START study, the correlation between quality of life and dysfunctional coping was 0.31; 105 people with dementia were needed to detect this association with 90% power and 5% significance.^18^ Adjustments were allowed for the cluster effect of care teams (the estimated mean size of care team was 40 residents with dementia; the intra-cluster correlation was 0.075^19^), the impact of confounding (variance inflation factor = 2) and predicted withdrawal rate (based on a withdrawal rate of 30% per year), the correlation between repeated measurements (from START data, 0.75) and inflation due to intention-to-measure interactions between different groups. As a result, the sample size (and therefore the recruitment target) was calculated as 1734 participants, assuming 20 residents with dementia recruited from 87 clusters.

### Quantitative variables

The CMAI is a 29-item questionnaire rating the frequency (from 1–7) of agitation behaviours common in dementia over a 2 week period. A score of 29 indicates no agitation, and one of >45 indicates clinically significant agitation. There are four subscales of agitation: aggressive behaviour (e.g. hitting), verbally agitated behaviour (e.g. constant requests for attention), physically non-aggressive behaviour (e.g. pacing or aimless wandering) and hiding/hoarding (see Supplementary Table 1 available at https://doi.org/10.1192/bjo.2021.21 for the factor structure). The CDR assesses function and cognition in six domains with a global impairment score. Both the CMAI and CDR are accepted as valid and reliable measures.^20,21^

Antipsychotics, antidepressants, anxiolytics/hypnotics and analgesics, as per the British National Formulary,^5^ were considered relevant. Only participants with both medication data and CMAI data were included in the medication analyses.

### Statistical method

We explored the study population at baseline using descriptive analyses. The total number and percentage (with 95% confidence intervals) of participants prescribed each drug type and prescription type (regular, PRN or both) were reported at each study visit. We reported the median dose, and the number of study visits (range 1–3; baseline and/or 4 months and/or 12 months) participants were prescribed each drug, excluding those who withdrew (for any reason, because inclusion would increase the number of false negatives of prescriptions that were stopped).

If residents were prescribed a drug class as PRN, then we reported how many residents were offered and administered at least one dose over a 2 week period, and the median number of doses (and the interquartile range; IQR) received per week.

All instances of missing data were described. In the case of items missing from the CMAI (and where researchers were unable to produce a total sum score), the data were visually assessed to consider whether missingness appeared random. If deemed random, the mean response of the available items was calculated and replaced the missing items (person mean imputation).^22^ This method was only used where more than 50% of the questionnaire was completed (questionnaires where more than 50% of items were missing were omitted from the analysis).

We used a multilevel linear regression model (clustered by care home) to explore, at baseline, the effects of care home factors on the prescription and administration of psychotropic drugs. Prior to analysing care home effects, we ran preliminary analyses (also clustered by care home) to identify potential predictor variables. The selected variables were age, sex, dementia diagnosis and severity, level of agitation (CMAI total score), ethnicity and first language. We conducted multilevel univariate logistic regression analyses to measure associations between whether or not the participant was prescribed a psychotropic at baseline and each potential predictor variable.

We compared psychotropic prescriptions and administration prevalence (as binary [prescribed/not prescribed; administered/not administered] variables) between resident characteristics. Before starting these tests, we conducted a sensitivity analysis to determine any differences in baseline factors between residents who had died during the study compared with those who were still alive (and still participating). A chi-squared test was used for binary variables, and *t*-tests were used for continuous variables, with missingness (resulting from death) at each study visit as an outcome. We also tested the data distribution to determine the most appropriate test. Mann–Whitney U-tests were used for nonparametric data. Any baseline variables that predicted missingness were included as independent variables in the models, as were resident factors if there was a clinical reason to do so.

Separate multilevel logistic regression models were used to explore the effects of age, sex, dementia severity (mild, moderate, severe) and agitation behaviours (whether agitation was clinically significant, CMAI score, agitation subtypes) on whether the drug was prescribed and, in the case of PRN drugs, administered. The data were analysed as longitudinal data, clustered by care home and repeated measures (baseline, 4 months, 12 months) within participants. Odds ratios (ORs) or regression coefficients with 95% confidence intervals are reported.

## Results

### Description of care homes and participants

At baseline there were 1454 residents with medication data, from 86 care homes. There were 50 (58.1%) nursing homes and 36 (41.9%) residential care homes. Sociodemographic data for residents are reported in Table 1; care home data are detailed in Supplementary Table 2. The majority (67.7%) of the study population were female, and the mean age was 84.9 (s.d. = 8.6). Severe dementia was more prevalent (37.7%) than moderate (32.7%) or mild dementia (29.6%). There were 574 (40.3%) residents with clinically significant agitation. At 12 months, 856 residents were still participating. See Supplementary Fig. 1 for a flow diagram of study participation and missingness.

**Table 1:** Resident characteristics at baseline (*n* = 1454)

Characteristic	*n* (%)
Female	985 (67.7)
Mean age ± s.d.	84.9 ± 8.6
Diagnosed dementia	1231 (84.7)
Dementia severity (CDR) (*n* = 1417)	
Mild	419 (29.6)
Moderate	464 (32.7)
Severe	534 (37.7)
CMAI subtypes (*n* = 1425)	
Clinically significant agitation (>45)	574 (40.3)
Aggressive behaviours	855 (60.0)
Physically non-aggressive behaviours	894 (62.7)
Verbally agitated	857 (60.1)
Hiding/hoarding	233 (16.4)

CDR, Clinical Dementia Rating; CMAI, Cohen–Mansfield Agitation Inventory.

### Prescribing prevalence and PRN administration

Table 2 lists the drug classes and prescription prevalence at baseline (see Supplementary Table 3 for 4 and 12 month and individual drug data). Prevalence rates reported here are at baseline and, unless otherwise specified, prescribing levels were stable throughout the study (see Supplementary Table 3 for details). Psychotropic drugs (regular or PRN) were prescribed to 822 participants (57.7%). Antidepressants were the most commonly prescribed class of psychotropic drug, prescribed to 578 residents (40.6%). The most commonly prescribed psychotropic drugs were citalopram (15.2%) and mirtazapine (11.2%). Four residents were prescribed PRN antidepressants.

**Table 2:** Prescribing prevalence of psychotropics and psychotropic drug classes at baseline (*n* = 1425)

Drug/drug type (WHO ATC code)	Total	Regular only	PRN only	Both regular and PRN
	*n* (%) [95% CI]			
Any psychotropic (N05A/N06B/N05C/N06A)	822^a^ (57.7%) [55.1–60.2]	655 (46.0%) [43.4–48.6]	64 (4.5%) [3.5–5.7]	102 (7.2%) [5.9–8.6]
Antipsychotics (N05A)	246 (17.3%) [15.4–19.3]	219 (15.4%) [13.6–17.3]	19 (1.3%) [0.9–2.1]	8 (0.6%) [0.3–1.1]
Anxiolytics/hypnotics (N05B/N05C)	310^a^ (21.8%) [19.7–24.0]	160 (11.2%) [9.7–13.0]	118 (8.3%) [7.0–9.8]	31 (2.2%) [5.9–8.6]
Antidepressants (N06A)	578^a^ (40.6%) [38.0–43.1]	573 (40.2%) [37.7–42.8]	3 (0.2%) [0.1–0.6]	1 (0.1%) [0.0–0.4]

WHO ATC, World Health Organisation Anatomical Therapeutic Chemical.

a. Total *n* does not match sum of last three columns because for one prescription it was not clear whether the prescription was regular or PRN.

Anxiolytics and hypnotics were prescribed to 310 residents (21.8%); 191 (13.4%) participants received regular prescriptions and 149 (10.5%) of participants had PRN drugs prescribed. The most commonly prescribed drugs in this class were lorazepam (prescribed to 8.4% of participants) and zopiclone (also prescribed to 8.4% of participants). A third of residents (32.7% [95% CI 30.3–35.2]) were prescribed antipsychotic and/or anxiolytic/hypnotic drugs. For all three subtypes of psychotropic, the median prescription duration (excluding withdrawn residents) was at least 1 year, i.e. three study visits.

At baseline, 14 days of PRN dose data for anxiolytics/hypnotics were available for 118 (79.2%) residents. Of those, 71 (60.2%) residents were offered and 62 (52.5%) were administered at least one anxiolytic/hypnotic drug. For residents who were administered a PRN anxiolytic/hypnotic, the median number of doses per resident per week was 5 (IQR, 2, 7).

Antipsychotics were least commonly prescribed, to 17.3% of participants. The most prevalent antipsychotic prescription was risperidone (prescribed to 7.4% of participants), followed by quetiapine (4.1%). Antipsychotic drugs were more commonly prescribed as regular prescriptions rather than PRN (15.9% *v*. 1.9%). There were 246 residents prescribed antipsychotics; 232 (94.3% [90.6–96.6]) residents were prescribed one antipsychotic, and 14 (5.7% [3.4–9.4]) were prescribed two antipsychotics.

There were 14 days of PRN dose data for antipsychotics available for 16 (59.3%) residents. Of those, 10 (62.5%) residents prescribed PRN were offered it and six (37.5%) residents received at least one PRN antipsychotic dose. For residents who were administered a PRN drug, the median number of doses per resident per week was 3 (IQR, 2–7).

Most PRN psychotropic prescriptions did not have an indication recorded. Where one was recorded, the majority of the reasons for prescribing antipsychotics and benzodiazepines were indicative of a behaviour associated with BPSD, for example, agitation or distress/anxiety (see Supplementary Table 4).

### Association between resident and care home factors and psychotropic drug use

Table 3 details the models describing the associations between resident and care home factors and use of each class of psychotropic. Female residents were prescribed more antidepressants (OR = 1.35, 95% CI: 1.14–1.59) and males were more likely to be prescribed anxiolytics/hypnotics (OR = 0.69, 95% CI: 0.57–0.84).

**Table 3:** Association between resident and care home factors and drug classes at baseline (*n* = 1425)

	Antipsychotics	Antidepressants	Anxiolytics/hypnotics
Resident factors			
Sex^a^ (Ref = Male)	0.95 (0.77, 1.18)	**1.35** (**1.14, 1.59)**^b^	**0.69** (**0.57, 0.84)**
Age^c^ (coef.)	**−0.04** (**−0.05, −0.03)**	**−0.03** (**-0.04, −0.02)**	**−0.04** (**−0.05, −0.03)**
Dementia severity^a^ (Ref = Mild)			
Moderate	1.01 (0.77, 1.32)	0.92 (0.76, 1.12)	1.10 (0.85, 1.43)
Severe	1.16 (0.90, 1.51)	**0.60** (**0.49, 0.73)**	**1.39** (**1.06, 1.78)**
Agitation^d^			
Clinically significant agitation	**2.00** (**1.64, 2.45)**	1.14 (0.98, 1.33)	**2.81** (**2.31, 3.40)**
Aggressive	1.01 (0.99, 1.02)	1.00 (0.99, 1.01)	1.01 (0.99, 1.02)
Physically non-aggressive	**1.04** (**1.02, 1.06)**	1.00 (0.98, 1.01)	**1.08** (**1.06, 1.09)**
Verbally agitated	**1.04** (**1.02. 1.06)**	**1.04** (**1.03, 1.06)**	**1.02** (**1.00, 1.04)**
Hiding/hoarding	**0.92** (**0.88, 0.97)**	0.97 (0.94, 1.01)	0.97 (0.93, 1.02)
Nursing homes	1.64 (0.99, 2.70)	1.12 (0.85, 1.48)	**2.23** (**1.36, 3.65)**
Ownership (Ref = Private)			
Charity	1.31 (0.70, 2.43)	1.01 (0.71, 1.44)	1.29 (0.70, 2.37)
Council/local authority	3.85 (1.25, 11.91)	0.75 (0.35, 1.64)	1.18 (0.35, 3.94)
Dementia registered	1.84 (0.66, 5.16)	1.04 (0.61, 1.78)	2.21 (0.80, 6.09)
Dementia specialist	1.10 (0.68, 1.78)	0.99 (0.75, 1.31)	1.38 (0.86, 2.20)
Number of beds	1.00 (0.99, 1.01)	1.00 (0.99, 1.00)	0.99 (0.98, 1.00)
CQC rating (Ref = Outstanding)			
Good	1.84 (0.74, 4.57)	1.06 (0.65, 1.71)	1.26 (0.54, 2.92)
Requires improvement	1.61 (0.57, 4.55)	1.32 (0.75, 2.33)	1.11 (0.42, 2.93)
Inadequate	1.57 (0.24, 10.15)	0.69 (0.23, 2.10)	0.17 (0.01, 2.06)

CQC, Care Quality Commission.

a. Controlling for age and CMAI total.

b. Significant results are shown in bold.

c. Controlling for gender.

d. Controlling for age, gender, and all CMAI factors.

Residents with severe dementia were less likely to be prescribed antidepressants (OR = 0.60, 95% CI: 0.49–0.73) and more likely to be prescribed anxiolytics/hypnotics (OR = 1.39, 95% CI: 1.06–1.78).

Antipsychotics were more likely to be prescribed to residents with clinically significant agitation (OR = 2.00, 95% CI: 1.64–2.45) and anxiolytics/hypnotics (OR = 2.81, 95% CI: 2.31–3.40). There was a positive association between verbal agitation and all drug classes, and between physically non-aggressive agitation and antipsychotics and anxiolytics/hypnotics. The only care home factor that was associated with psychotropic drug use was nursing homes: residents in nursing homes were prescribed more anxiolytics/hypnotics (OR = 2.23, 95% CI: 1.36–3.65). Table 3 describes all associations.

## Discussion

### Key results

At baseline, approximately one in six residents were prescribed an antipsychotic drug, and around one-fifth of residents were prescribed an anxiolytic/hypnotic drug; a third of residents were prescribed either an antipsychotic or an anxiolytic/hypnotic. Where indications were reported, the majority of antipsychotics and benzodiazepines were prescribed for BPSD; prescribing may therefore frequently contradict recommendations.^1,6^ It is concerning that antipsychotics and anxiolytics/hypnotics were prescribed for durations that long exceed guidelines, over the whole study, so for at least 1 year. Despite guidance proscribing this,^1,6^ there were 14 residents prescribed two antipsychotics. Two-fifths of residents were prescribed antidepressants, including PRN prescriptions; there is no indication to prescribe antidepressants PRN. Anxiolytics/hypnotics were the most commonly prescribed PRN medication and were prescribed for about 10.5% of residents, but antipsychotics were prescribed PRN for only about 2%. People who were given PRN medication usually had it several days of the week.

PRN psychotropics are frequently prescribed in care homes. There has been little analysis of how often these drugs are administered or of factors that influence their use. The data presented here are a useful contribution to the existing literature but also demonstrate the importance of collecting PRN medication data in future studies, for researchers and care home prescribers.

Some groups were more likely to be prescribed psychotropic drugs: residents with clinically significant agitation were prescribed twice as many antipsychotics and over twice as many anxiolytics/hypnotics. There was a positive association between verbal agitation and all drug classes. Females were more likely to be prescribed antidepressants, and males were more likely to be prescribed anxiolytics/hypnotics.

### Limitations

The data did not include drug indications for regular prescriptions, and the majority of PRN prescriptions were not accompanied by an indication, or medical or mental comorbidities, so it was not possible to assess the appropriateness of the prescription. As an example, antidepressants may be prescribed for neuropathic pain. This is a limitation of the study, and the data also showed that indications for medication are often poorly documented in care homes. In addition, when assessing factors associated with psychotropic use, comparing average doses may be a better measure than number of doses. A further limitation is that the prescription duration data assume that if a drug was prescribed at all (three) study visits then it was prescribed as 1 year of continuous prescription; however, this does not account for whether prescriptions were stopped and started between study visits.

Finally, there were no data on how medicines were managed and reviewed. In care homes where PRN prescriptions are more personalised, PRN administration may also be expected to be higher, because there are fewer ‘blanket’ PRN prescriptions. Information regarding support arrangements may have explained some of the observed variation between homes; its omission limits how we can interpret these data. Prescribers can influence how psychotropics are used, and data measuring medication management (for example, frequency of medication reviews and who is involved) within the care home would be interesting to include in future studies. It is also important to ensure that the data collected can appropriately measure care home differences and can capture the quality of interactions, information-sharing and burden of care.

### Interpretation

Prescriptions of psychotropics to care home residents with dementia often have harmful side-effects but are frequent and prolonged. These residents are prescribed more psychotropics than people with dementia in the community,^3,9^ possibly because people are often admitted to care homes with BPSD as these symptoms predict breakdown of care. In the context of national guidelines advising that BPSD should only be pharmacologically treated in cases of extreme distress or risk, and for short periods only, we show that psychotropic prescribing in care homes is outside recommendations. Similar results were found in a study assessing associations between the launch of England's National Dementia Strategy and antipsychotic prescribing in care homes.^11^ Excessive prescribing duration of psychotropics has also been found in people with dementia who live outside care homes.^7^ A MARQUE study on prevalence and associations with agitation concluded that staff should be supported to provide personalised approaches in response to agitation and to enable them to identify the cause through improved communication and engagement.^12^ Psychotropics may be a useful tool in managing behaviours when prescribed alongside personalised psychosocial interventions.

Residents with clinically significant agitation were twice as likely to be prescribed antipsychotics and even more likely to be prescribed anxiolytics or hypnotics. Although 40% of residents had clinically significant agitation, the behaviours reported were at the time of our survey often neither severe nor risky, and so, according to guidelines, psychotropics were not indicated. Further, benzodiazepines and hypnotics including zopiclone were frequently prescribed, despite warnings advising against use in this population.^23^ Sedating benzodiazepines may be prescribed as a replacement for antipsychotics. It is vital that if policies advocate reducing use of a certain drug, or drug class, they should also anticipate potential substitutions and provide appropriate guidance.^9,24^

Verbally agitated behaviour was associated with increased use of all drug classes (controlling for other agitation factors). It may be that aggressive behaviours are being effectively managed by psychotropics but verbally agitated behaviours are not. Verbal agitation may be caused by pain;^25^ in these cases, psychotropics will not address the cause of the behaviour, and pain may not be treated effectively.

Males were prescribed significantly more anxiolytics and hypnotics than females. Given that guidelines advocate stopping antipsychotics, it may be that agitation in males is now being treated with this drug class. Aggression (particularly from males) may be seen as more threatening, which may explain higher prescribing in these two (possibly overlapping) groups.^26^ A study in German and Austrian care homes also found that males were prescribed more anxiolytics;^27^ however, this sex difference is not always observed.^28^

A large proportion of residents were prescribed antidepressants, despite current evidence of lack of efficacy.^29^ Although the higher boundary of estimates of depression in people with dementia is similar to our prescribing rate of approximately 40%, prevalence of clinically significant depressive symptoms is lower, at around 10–35%.^30,31^ There are mixed findings regarding sex differences in depression prevalence and antidepressant treatment in care homes, and although women generally are more likely to be depressed, this appears to be less so in older age groups.^32,33^

Citalopram was the most frequently prescribed antidepressant in this care home cohort, whereas in the community, sertraline was the most commonly prescribed antidepressant.^34^ Citalopram may be prescribed more frequently for a number of reasons: sertraline may not be effective in the treatment of depression in people with Alzheimer's disease;^35^ and sertraline needs to follow a titration schedule, whereas citalopram may be prescribed off-licence to manage agitation. However, there are concerns regarding adverse cardiac effects that should be considered, and which may limit its efficacy for agitation.^36^

### Generalisability

The MARQUE study offers the strength of a unique, large and robust data-set from across England, with similar demographics to those of international studies.^37^ The care homes were recruited from across England, and the number of recruited residents represents the largest prospective care home study to date. It is important to include a large sample size to capture the heterogeneity of care home residents. Dementia is underdiagnosed in this population, so the ability to include those with probable dementia was a strength; if a clinical dementia diagnosis had been a criterion, a large proportion of residents would have been excluded.^37,38^ The study benefited from wide inclusion criteria; all eligible residents with cognitive impairment (or their next of kin) were contacted, reducing selection bias and increasing external validity.

### Implications

Recent policy drives do not seem to have consistently improved psychotropic prescribing. Psychotropics, in particular antipsychotic, anxiolytic and hypnotic drugs, continue to be prescribed to residents with symptoms that may not warrant pharmacological intervention, and for longer durations than recommended. Psychotropic overuse may occur for a number of reasons. Care homes may be struggling to deliver effective non-pharmacological interventions, and medication reviews may not be completed regularly or effectively.^39^ Care homes and general practitioners (GPs) could be better supported by geriatricians, community pharmacists, enhanced care teams or care home liaison nurses.^40,41^ In 2018, the Royal Pharmaceutical Society stated that pharmacists should work alongside GPs to provide regular medication reviews in care homes, and in response NHS England intend to recruit more pharmacists.^42,43^

The recent guidelines published by the Alzheimer's Society regarding doses, indications and prescription duration could provide much-needed specific guidance for prescribers to follow.^1^ More care homes are using electronic systems to record medical and care data. These systems should be developed to flag inappropriate prescriptions, such as benzodiazepines prescribed for more than 4 weeks. These systems could also be designed to ensure that all information is recorded, including indications. Hence, prescriptions will be less likely to ‘slip through the net’ and the effectiveness of the prescription and any potential side-effects can be reviewed regularly.

## Data Availability

The data that support the findings of this study are available from the corresponding author, E.L.S., upon reasonable request.

## Footnotes

1 Part of this paper was presented in F.L.F.'s PhD thesis: La Frenais F. A longitudinal study exploring the use of analgesic medication in English care home residents with dementia. *Doctoral thesis (PhD)*, University College London, 2018.

## Supplementary material

For supplementary material accompanying this paper visit http://dx.doi.org/10.1192/bjo.2021.21. click here to view supplementary material
