# Supplementary material for: Psychotropic prescribing for English care home residents with dementia compared with national guidance: findings from the MARQUE national longitudinal study
Source: BJPsych Open. 2021 Sep 15;7(5):e169. doi: 10.1192/bjo.2021.21 (PMC8485345; doi:10.1192/bjo.2021.21)
Supplement: Supplementary file 1 [file S2056472421000211sup001.docx]

Supplementary table 1: Factor structure of Cohen-Mansfield Agitation Inventory

| Factor | Behaviors |
| --- | --- |
| Aggressive behavior | Hitting, kicking, pushing, scratching, grabbing, cursing or verbal aggression, hurting self or other, biting, spitting, throwing things, tearing things or destroying property, screaming |
| Physically nonaggressive behavior | Pacing or aimless wandering, inappropriate undressing or disrobing, performing repetitive mannerisms, trying to get to a different place, handling things inappropriately, general restlessness |
| Verbally agitated behavior | Constant requests for attention, repetitive sentences or questions, complaining, negativism |
| Hiding/hoarding behaviors | Hiding, hoarding |
| Excluded factors | Low occurrence: intentional falling, verbal sexual advances, physical sexual advances  Did not load: strange noises, eating or drinking inappropriate substances |

Supplementary table 2: Consented care home characteristics (N=86)

| **Type** | **N** | **%** | **National average^1^, %** |
| --- | --- | --- | --- |
| Nursing | 50 | 58.1 | 26.0^1^ |
| Residential | 36 | 41.9 | 74.0^1^ |
| **Provider** |  |  |  |
| Charity/Voluntary | 15 | 17.4 | 20.7 |
| Council/Local authority | 2 | 2.3 | 4.0 |
| Independent/Private | 68 | 79.1 | 75.0 |
| NHS | 1 | 1.2 | 0.3 |
| **Registration** |  |  |  |
| Dementia specialist | 76 | 87.4 | 46.1 |
| Dementia registered | 29 | 33.3 | 15.0 |
| **Care Quality Commission rating** | |  |  |
| Outstanding | 6 | 7.1 | 0.6 |
| Good | 59 | 69.4 | 49.6 |
| Requires improvement | 18 | 21.2 | 45.1 |
| Inadequate | 2 | 2.4 | 4.8 |

^1^Care quality commission 2016. The state of health care and adult social care in England 2015/16 UK.

Supplementary figure 1: STROBE diagram of study participant recruitment


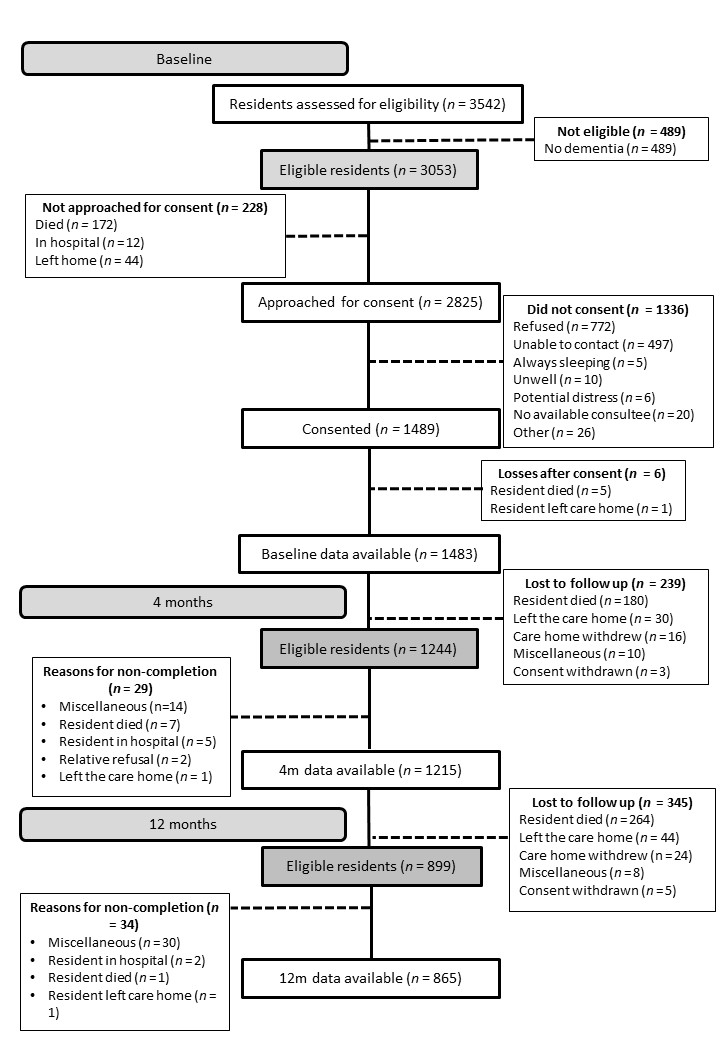


Supplementary table 3: Prescribing prevalence of psychotropic classes and drugs at each study visit, mean daily dose, and indication (baseline (n=1425), 4-month (n=1215), and 12-month (n=856))

|  | **Study visit** | **Total**  N (%)  [95% CI] | **Regular only**  N (%)  [95% CI] | **PRN only**  N (%)  [95% CI] | **Both regular + PRN**  N (%)  [95% CI] | **Daily dose (mg)**  Mean (SD)  range |
| --- | --- | --- | --- | --- | --- | --- |
| **Psychotropics** | Baseline | 822 (57.7%)  [55.1-60.2] | 655 (46.0%)  [43.4-48.6] | 64 (4.5%)  [3.5-5.7] | 102 (7.2%)  [5.9-8.6] | na |
|  | 4-month | 690 (56.8%)  [54.0-59.6] | 540 (44.4%)  [41.7-47.3] | 47 (3.9%)  [2.9-5.1] | 103 (8.5%)  [7.0-10.2] | na |
|  | 12-month | 491 (57.4%)  [54.0-60.6] | 371 (43.3%)  [40.0-46.7] | 46 (5.4%)  [4.0-7.1] | 74 (8.6%)  [6.9-10.7] | na |
|  | Median (IQR) study visits that drug is prescribed: 2 (2, 3) (inc. withdrawn residents), 3 (2, 3) (exc. withdrawn residents) | | | | | |
| **Anxiolytics and hypnotics** | Baseline | 310 (21.8%)  [19.7-24.0] | 160 (11.2%)  [9.7-13.0] | 118 (8.3%)  [7.0-9.8] | 31 (2.2%)  [5.9-8.6] | na |
|  | 4-month | 276 (22.7%)  [20.4-25.2] | 139 (11.4%)  [9.8-13.4] | 109 (9.0%)  [7.5-10.7] | 28 (2.3%)  [1.6-3.3) | na |
|  | 12-month | 195 (22.8%)  [20.1-25.7] | 87 (10.2%)  [8.3-12.4] | 87 (10.2%)  [8.3-12.4] | 21 (2.5%)  [1.6-3.7] | na |
|  | Median (IQR) study visits that drug is prescribed: 3 (2, 3) (inc. withdrawn residents), 3 (2, 3) (exc. withdrawn residents) | | | | | |
| Buspirone | Baseline | 3 (0.2%)  [0.1-0.7] | 2 (0.1%)  [0.0-0.6] | 1 (0.1%)  [0.0-0.5] | 0 (0%) | 11.7 (5.8)  5-15 |
|  | 4-month | 2 (0.2%)  [0.0-0.6] | 2 (0.2%)  [0.0-0.6] | 0 (0%) | 0 (0%) | 10.0 (7.1)  5-15 |
|  | 12-month | 1 (0.1%)  [0.0-0.8] | 1 (0.1%)  [0.0-0.8] | 0 (0%) | 0 (0%) | 5.0 (0.0) |
|  | Median (IQR) study visits that drug is prescribed: 3 (2, 3) (inc. withdrawn residents), 3 (3, 3) (exc. withdrawn residents) | | | | | |
| Clobazam | Baseline | 1 (0.1%)  [0.0-0.5] | 0 (0%) | 1 (0.1%)  [0.0-0.5] | 0 (0%) | 10 (0) |
|  | 4-month | 0 (0%) | 0 (0%) | 0 (0%) | 0 (0%) | na |
|  | 12-month | 0 (0%) | 0 (0%) | 0 (0%) | 0 (0%) | na |
|  | Median (IQR) study visits that drug is prescribed: 1 (N/A) (inc. withdrawn residents), 1 (N/A) (exc. withdrawn residents) | | | | | |
| Clonazepam | Baseline | 21 (1.5%)  [1.0-2.3] | 17 (1.2%)  [0.7-1.9] | 4 (0.3%)  [0.1-0.8] | 0 (0%) | 0.7 (0.4)  0.25-1.5 |
|  | 4-month | 15 (1.2%)  [0.7-2.0] | 14 (1.2%)  [0.7-1.9] | 1 (0.1%)  [0.0-0.6] | 0 (0%) | 0.8 (0.5)  0.25-2 |
|  | 12-month | 14 (1.6%)  [1.0-2.7] | 12 (1.4%)  [0.8-2.5] | 2 (0.2%)  [0.1-0.9] | 0 (0%) | 1.0 (0.7)  0.25-2.7 |
|  | Median (IQR) study visits that drug is prescribed: 3 (1, 3) (inc. withdrawn residents), 3 (2, 3) (exc. withdrawn residents) | | | | | |
| Diazepam | Baseline | 46 (3.2%)  [2.4-4.3] | 17 (1.2%)  [0.7-1.9] | 27 (1.9%)  [1.3-2.8] | 2 (0.1%)  [0.0-0.6] | 5.2 (3.9)  0.5-20 |
|  | 4-month | 43 (3.5%)  [2.6-4.7] | 13 (1.1%)  [0.6-1.8] | 28 (2.3%)  [1.6-3.3] | 2 (0.2%)  [0.0-0.7] | 4.6 (2.7)  2-12 |
|  | 12-month | 26 (3.0%)  [2.1-4.4] | 8 (0.9%)  [0.5-1.9] | 18 (2.1%)  [1.3-3.3] | 0 (0%) | 6.2 (5.3)  1-20 |
|  | Median (IQR) study visits that drug is prescribed: 3 (2, 3) (inc. withdrawn residents), 3 (2, 3) (exc. withdrawn residents) | | | | | |
| Lorazepam | Baseline | 120 (8.4%)  [7.1-10.0] | 38 (2.7%)  [1.9-3.6] | 80 (5.6%)  [4.5-6.9] | 2 (0.1%)  [0.0-0.6] | 1.5 (0.9)  0.5-4 |
|  | 4-month | 112 (9.2%)  [7.7-11.0] | 39 (3.2%)  [2.4-4.4] | 69 (5.7%)  [4.5-7.1] | 4 (0.3%)  [0.1-0.9) | 1.5 (1.2)  0.5-9 |
|  | 12-month | 80 (9.3%)  [7.6-11.5] | 22 (2.6%)  [1.7-3.9] | 56 (6.5%)  [5.1-8.4] | 2 (0.2%)  [0.1-0.9] | 1.5 (0.8)  0.5-4 |
|  | Median (IQR) study visits that drug is prescribed: 3 (2, 3) (inc. withdrawn residents), 3 (2.5, 3) (exc. withdrawn residents) | | | | | |
| Lormetazepam | Baseline | 1 (0.1%)  [0.0-0.5] | 1 (0.1%)  [0.0-0.5] | 0 (0%) | 0 (0%) | 1.0 (0) |
|  | 4-month | 1 (0.1%)  [0.0-0.6] | 1 (0.1%)  [0.0-0.6] | 0 (0%) | 0 (0%) | 1.0 (0) |
|  | 12-month | 1 (0.1%)  [0.0-0.6] | 1 (0.1%)  [0.0-0.6] | 0 (0%) | 0 (0%) | 1.0 (0) |
|  | Median (IQR) study visits that drug is prescribed: 3 (N/A) (inc. withdrawn residents), 3 (N/A) (exc. withdrawn residents) | | | | | |
| Midazolam | Baseline | 23 (1.6%)  [1.1-2.4] | 0 (0%) | 22 (1.5%)  [1.0-2.3] | 0 (0%) | 34.5 (22.2)  2.5-60 |
|  | 4-month | 25 (2.1%)  [1.4-3.0] | 1 (0.1%)  [0.0-0.5] | 24 (1.7%)  [1.1-2.5] | 0 (0%) | 78.8 (63.5)  20-240 |
|  | 12-month | 23 (2.7%)  [1.8-4.0] | 4 (0.5%)  [0.1-1.2] | 19 (2.2%)  [1.3-3.4] | 0 (0%) | 43.0 (26.3)  20-120 |
|  | Median (IQR) study visits that drug is prescribed: 3 (3, 3) (inc. withdrawn residents), 3 (1, 3) (exc. withdrawn residents) | | | | | |
| Nitrazepam | Baseline | 4 (0.3%)  [0.1-0.7] | 4 (0.3%)  [0.1-0.7] | 0 (0%) | 0 (0%) | 5.9 (1.2)  5-7.5 |
|  | 4-month | 5 (0.4%)  [0.2-1.0] | 5 (0.4%)  [0.2-1.0] | 0 (0%) | 0 (0%) | 5.0 (1.8)  2.5-7.5 |
|  | 12-month | 4 (0.4%)  [0.1-1.2] | 4 (0.4%)  [0.1-1.2] | 0 (0%) | 0 (0%) | 5.6 (1.3)  5-7.5 |
|  | Median (IQR) study visits that drug is prescribed: 2 (1, 3) (inc. withdrawn residents), 2.5 (1.5, 3) (exc. withdrawn residents) | | | | | |
| Oxazepam | Baseline | 5 (0.4%)  [0.1-0.8] | 1 (0.1%)  [0.0-0.5] | 3 (0.2%)  [0.1-0.7] | 1 (0.1%)  [0.0-0.5] | 17.5 (9.6)  10-30 |
|  | 4-month | 4 (0.3%)  [0.1-0.9] | 1 (0.1%)  [0.0-0.6] | 3 (0.2%)  [0.1-0.8] | 0 (0%) | 20 (0.0) |
|  | 12-month | 3 (0.4%)  [0.1-1.1] | 1 (0.1%)  [0.0-0.8] | 2 (0.2%)  [0.1-0.9] | 0 (0%) | 15.0 (7.1)  10-20 |
|  | Median (IQR) study visits that drug is prescribed: 3 (3, 3) (inc. withdrawn residents), 3 (2.5, 3) (exc. withdrawn residents) | | | | | |
| Temazepam | Baseline | 14 (1.0%)  [0.6-1.7] | 13 (0.9%)  [0.5-1.6] | 1 (0.1%)  [0.0-0.5] | 0 (0%) | 12.1 (6.4)  5-20 |
|  | 4-month | 11 (0.9%)  [0.5-1.6] | 11 (0.9%)  [0.5-1.6] | 0 (0%) | 0 (0%) | 12.3 (6.5)  5-20 |
|  | 12-month | 6 (0.7%)  [0.3-1.6] | 6 (0.7%)  [0.3-1.6] | 0 (0%) | 0 (0%) | 10.8 (4.9)  5-20 |
|  | Median (IQR) study visits that drug is prescribed: 2.5 (2, 3) (inc. withdrawn residents), 3 (3, 3) (exc. withdrawn residents) | | | | | |
| Zolpidem | Baseline | 5 (0.4%)  [0.1-0.8] | 5 (0.4%)  [0.1-0.8] | 0 (0%) | 0 (0%) | 5 (0) |
|  | 4-month | 5 (0.4%)  [0.2-1.0] | 5 (0.4%)  [0.2-1.0] | 0 (0%) | 0 (0%) | 5 (0) |
|  | 12-month | 4 (0.5%)  [0.1-1.2] | 3 (0.4%)  [0.1-1.0] | 1 (0.1%)  [0.0-0.6] | 0 (0%) | 5.0 (0.0) |
|  | Median (IQR) study visits that drug is prescribed: 2 (2, 3) (inc. withdrawn residents), 2 (2, 3) (exc. withdrawn residents) | | | | | |
| Zopiclone | Baseline | 119 (8.4%)  [7.0-9.9] | 100 (7.0%)  [5.8-8.5] | 18 (1.3%)  [0.8-2.0] | 1 (0.1%)  [0.0-0.5] | 5.2 (2.0)  3.5-15 |
|  | 4-month | 98 (8.1%)  [6.7-9.7] | 82 (6.7%)  [5.5-8.3] | 15 (1.2%)  [0.7-2.0] | 1 (0.1%)  [0.0-0.5] | 4.9 (1.7)  3.75-7.5 |
|  | 12-month | 68 (7.9%)  [6.3-10.0] | 50 (5.8%)  [4.5-7.6] | 18 (2.1%)  [1.3-3.3] | 0 (0%) | 5.0 (1.8)  3.75-7.5 |
|  | Median (IQR) study visits that drug is prescribed: 2 (1, 3) (inc. withdrawn residents), 2.5 (2, 3) (exc. withdrawn residents) | | | | | |
| **Antidepressants** | Baseline | 578 (40.6%)  [38.0-43.1] | 573 (40.2%)  [37.7-42.8] | 3 (0.2%)  [0.1-0.6] | 1 (0.1%)  [0.0-0.4] | na |
|  | 4-month | 485 (40.0%)  [37.2-42.7] | 482 (39.7)  [37.0-42.5] | 2 (0.2%)  [0.0-0.7] | 1 (0.1%)  [0.0-0.6] | na |
|  | 12-month | 337 (39.4%)  [36.1-42.7] | 335 (39.1%)  [35.9-42.5] | 2 (0.2%)  [0.1-0.9] | 0 (0%) | na |
|  | Median (IQR) study visits that drug is prescribed: 2 (1.5, 3) (inc. withdrawn residents), 3 (2, 3) (exc. withdrawn residents) | | | | | |
| Amitriptyline | Baseline | 33 (2.3%)  [0.7-2.3] | 31 (2.2%)  [1.5-3.1] | 2 (0.1%)  [0.0-0.5] | 0 (0%) | 18.0 (18.3)  5-100 |
|  | 4-month | 30 (2.5%)  [1.7-3.5] | 30 (2.5%)  [1.7-3.5] | 0 (0%) | 0 (0%) | 19.0 (20.0)  5-100 |
|  | 12-month | 17 (2.0%)  [1.2-3.2] | 17 (2.0%)  [1.2-3.2] | 0 (0%) | 0 (0%) | 25.0 (25.7)  5-100 |
|  | Median (IQR) study visits that drug is prescribed: 2 (2, 3) (inc. withdrawn residents), 3 (2, 3) (exc. withdrawn residents) | | | | | |
| Citalopram | Baseline | 217 (15.2%)  13.5-17.2] | 217 (15.2%)  13.5-17.2] | 0 (0%) | 0 (0%) | 17.3 (9.1)  5-100 |
|  | 4-month | 172 (14.2%)  [12.2-16.2] | 172 (14.2%)  [12.2-16.2] | 0 (0%) | 0 (0%) | 17.6 (13.1)  5-160 |
|  | 12-month | 117 (13.7%)  [11.4-16.2] | 117 (13.7%)  [11.4-16.2] | 0 (0%) | 0 (0%) | 17.8 (9.6)  10-80 |
|  | Median (IQR) study visits that drug is prescribed: 2 (1, 3) (inc. withdrawn residents), 3 (2, 3) (exc. withdrawn residents) | | | | | |
| Duloxetine | Baseline | 9 (0.6%)  [0.3-1.2] | 9 (0.6%)  [0.3-1.2] | 0 (0%) | 0 (0%) | 45.0 (16.0)  30-60 |
|  | 4-month | 8 (0.7%)  [0.3-1.3] | 8 (0.7%)  [0.3-1.3] | 0 (0%) | 0 (0%) | 43.1 (18.7)  15-60 |
|  | 12-month | 6 (0.7%)  [0.3-1.5] | 6 (0.7%)  [0.3-1.5] | 0 (0%) | 0 (0%) | 48.3 (13.3)  30-60 |
|  | Median (IQR) study visits that drug is prescribed: 2 (1, 3) (inc. withdrawn residents), 2.5 (1, 3) (exc. withdrawn residents) | | | | | |
| Fluoxetine | Baseline | 31 (2.2%)  [1.5-3.1] | 31 (2.2%)  [1.5-3.1] | 0 (0%) | 0 (0%) | 23.0 (7.9)  10-40 |
|  | 4-month | 26 (2.1%)  [1.4-3.1] | 26 (2.1%)  [1.4-3.1] | 0 (0%) | 0 (0%) | 25.8 (13.9)  10-80 |
|  | 12-month | 13 (1.5%)  [0.8-2.6] | 13 (1.5%)  [0.8-2.6] | 0 (0%) | 0 (0%) | 21.5 (5.5)  20-40 |
|  | Median (IQR) study visits that drug is prescribed: 2 (2, 3) (inc. withdrawn residents), 3 (2, 3) (exc. withdrawn residents) | | | | | |
| Lofepramine | Baseline | 6 (0.7%)  [0.3-1.5] | 6 (0.7%)  [0.3-1.5] | 0 (0%) | 0 (0%) | 122.5 (75.9)  35-210 |
|  | 4-month | 5 (0.4%)  [0.1-1.0] | 5 (0.4%)  [0.1-1.0] | 0 (0%) | 0 (0%) | 70.0 (0.0) |
|  | 12-month | 2 (0.2%)  [0.0-0.8] | 2 (0.2%)  [0.0-0.8] | 0 (0%) | 0 (0%) | 140.0 (99.0)  70-210 |
|  | Median (IQR) study visits that drug is prescribed: 1 (1, 1) (inc. withdrawn residents), 1 (1, 1) (exc. withdrawn residents) | | | | | |
| Mirtazapine | Baseline | 159 (11.2%)  [9.6-12.9] | 159 (11.2%)  [9.6-12.9] | 0 (0%) | 0 (0%) | 28.0 (11.3)  7.5-45 |
|  | 4-month | 136 (11.2%)  [8.5-13.1] | 136 (11.2%)  [8.5-13.1] | 0 (0%) | 0 (0%) | 27.5 (12.1)  5-75 |
|  | 12-month | 94 (11.0%)  [8.9-13.3] | 94 (11.0%)  [8.9-13.3] | 0 (0%) | 0 (0%) | 28.9 (11.1)  7.5-45 |
|  | Median (IQR) study visits that drug is prescribed: 2 (1, 3) (inc. withdrawn residents), 3 (2, 3) (exc. withdrawn residents) | | | | | |
| Paroxetine | Baseline | 12 (0.8%)  [0.5-1.5] | 12 (0.8%)  [0.5-1.5] | 0 (0%) | 0 (0%) | 22.5 (6.2)  10-30 |
|  | 4-month | 10 (0.8%)  [0.4-1.5] | 10 (0.8%)  [0.4-1.5] | 0 (0%) | 0 (0%) | 23.3 (5.0)  20-30 |
|  | 12-month | 9 (1.1%)  [0.5-2.0] | 9 (1.1%)  [0.5-2.0] | 0 (0%) | 0 (0%) | 22.2 (6.7)  10-30 |
|  | Median (IQR) study visits that drug is prescribed: 3 (2.5, 3) (inc. withdrawn residents), 3 (3, 3) (exc. withdrawn residents) | | | | | |
| Phenelzine | Baseline | 1 (0.1%)  [0.0-0.5] | 1 (0.1%)  [0.0-0.5] | 0 (0%) | 0 (0%) | 30.0 (0.0) |
|  | 4-month | 1 (0.1%)  [0.0-0.5] | 1 (0.1%)  [0.0-0.5] | 0 (0%) | 0 (0%) | 30.0 (0.0) |
|  | 12-month | 0 (0%) | 0 (0%) | 0 (0%) | 0 (0%) | na |
|  | Median (IQR) study visits that drug is prescribed: 2 (2, 2) (inc. withdrawn residents), 2 (2, 2) (exc. withdrawn residents) | | | | | |
| Sertraline | Baseline | 81 (5.7%)  [4.6-7.0] | 80 (5.6)  [4.5-6.9] | 0 (0%) | 0 (0%) | 85.6 (67.8)  25-500 |
|  | 4-month | 75 (6.2%)  [4.9-7.7] | 75 (6.2%)  [4.9-7.7] | 0 (0%) | 0 (0%) | 77.8 (42.6)  [25-200] |
|  | 12-month | 64 (7.5%)  [5.8-9.4] | 64 (7.5%)  [5.8-9.4] | 0 (0%) | 0 (0%) | 75.0 (42.0)  25-200 |
|  | Median (IQR) study visits that drug is prescribed: 2 (1, 3) (inc. withdrawn residents), 3 (2, 3) (exc. withdrawn residents) | | | | | |
| Trazodone | Baseline | 62 (4.4%)  [3.4-5.5] | 60 (4.2%)  [3.3-5.4] | 2 (0.1%)  [0.0-0.6] | 0 (0%) | 92.8 (53.6)  25-250 |
|  | 4-month | 47 (3.9%)  [2.9-5.1] | 44 3.6%)  [2.7-4.8] | 2 (0.2%)  [0.0-0.7] | 1 (0.1%)  [0.0-0.5] | 92.1 (45.8)  10-200 |
|  | 12-month | 30 (3.5%)  P2.5-5.0] | 28 (3.3%)  [2.3-4.7] | 2 (0.2%)  [0.1-0.9] | 0 (0%) | 91.7 (47.9)  25-200 |
|  | Median (IQR) study visits that drug is prescribed: 2 (1, 3) (inc. withdrawn residents), 3 (1, 3) (exc. withdrawn residents) | | | | | |
| Venlafaxine | Baseline | 17 (1.2%)  [0.7-1.9] | 17 (1.2%)  [0.7-1.9] | 0 (0%) | 0 (0%) | 113.8 (53.7)  22-225 |
|  | 4-month | 16 (1.3%)  [0.8-2.1] | 16 (1.3%)  [0.8-2.1] | 0 (0%) | 0 (0%) | 124.2 (56.0)  37.5-225 |
|  | 12-month | 14 (1.6%)  [0.9-2.7] | 14 (1.6%)  [0.9-2.7] | 0 (0%) | 0 (0%) | 133.9 (52.4)  75-225 |
|  | Median (IQR) study visits that drug is prescribed: 3 (2, 3) (inc. withdrawn residents), 3 (2, 3) (exc. withdrawn residents) | | | | | |
| **Antipsychotics** | Baseline | 246 (17.3%)  [15.4-19.3] | 219 (15.4%)  [13.6-17.3] | 19 (1.3%)  [0.9-2.1] | 8 (0.6%)  [0.3-1.1] | na |
|  | 4-month | 209 (17.2%)  [15.2-19.4] | 189 (15.6%)  13.6-17.7] | 14 (1.2%)  [0.6-1.9] | 6 (0.5%)  [0.2-1.1] | na |
|  | 12-month | 158 (18.5%)  [16.0-21.2] | 136 (15.9%)  [13.6-18.5] | 14 (1.6%)  [1.0 -2.7] | 8 (0.9)  [0.5-1.9] | na |
|  | Median (IQR) study visits that drug is prescribed: 2 (2, 3) (inc. withdrawn residents), 3 (2, 3) (exc. withdrawn residents) | | | | | |
| Amisulpride | Baseline | 16 (1.1%)  [0.7-1.8] | 16 (1.1%)  [0.7-1.8] | 0 (0%) | 0 (0%) | 98.4 (97.7)  25-400 |
|  | 4-month | 11 (0.9%)  [0.5-1.6] | 10 (0.8%)  [0.4-1.5] | 0 (0%) | 1 (0.1%)  [0.0-0.5] | 87.5 (66.9)  25-200 |
|  | 12-month | 9 (1.1%)  [0.5-2.0] | 9 (1.1%)  [0.5-2.0] | 0 (0%) | 0 (0%) | 133.3 (136.4)  25-400 |
|  | Median (IQR) study visits that drug is prescribed: 2 (1, 3) (inc. withdrawn residents), 3 (2, 3) (exc. withdrawn residents) | | | | | |
| Aripiprazole | Baseline | 6 (0.4%)  [0.2-0.9] | 6 (0.4%)  [0.2-0.9] | 0 (0%) | 0 (0%) | 12.1 (7.5)  2.5-20 |
|  | 4-month | 5 (0.4%)  [0.1-1.0] | 5 (0.4%)  [0.1-1.0] | 0 (0%) | 0 (0%) | 14 (6.5)  5-20 |
|  | 12-month | 5 (0.6%)  [0.2-1.4] | 5 (0.6%)  [0.2-1.4] | 0 (0%) | 0 (0%) | 9.0 (4.2)  5-15 |
|  | Median (IQR) study visits that drug is prescribed: 2 (1, 3) (inc. withdrawn residents), 2.5 (1, 3) (exc. withdrawn residents) | | | | | |
| Chlorpromazine | Baseline | 1 (0.1)  [0.0-0.5] | 1 (0.1)  [0.0-0.5] | 0 (0%) | 0 (0%) | 179.0 (0.0) |
|  | 4-month | 1 (0.1%)  [0.0-0.5] | 1 (0.1%)  [0.0-0.5] | 0 (0%) | 0 (0%) | 175.0 (0.0) |
|  | 12-month | 0 (0%) | 0 (0%) | 0 (0%) | 0 (0%) | na |
|  | Median (IQR) study visits that drug is prescribed: 2 (2, 2) (inc. withdrawn residents), 2 (2, 2) (exc. withdrawn residents) | | | | | |
| Clozapine | Baseline | 2.0 (0.1%)  [0.0-0.6] | 2.0 (0.1%)  [0.0-0.6] | 0 (0%) | 0 (0%) | 275.0 (35.4)  250-300 |
|  | 4-month | 2 (0.2%)  [0.0-0.6] | 2 (0.2%)  [0.0-0.6] | 0 (0%) | 0 (0%) | 275.0 (35.4)  250-300 |
|  | 12-month | 1 (0.1%)  [0.0-0.6] | 1 (0.1%)  [0.0-0.6] | 0 (0%) | 0 (0%) | 250.0 (0.0) |
|  | Median (IQR) study visits that drug is prescribed: 2.5 (2, 3) (inc. withdrawn residents), 3 (3, 3) (exc. withdrawn residents) | | | | | |
| Flupentixol | Baseline | 4 (0.3%)  [0.1-0.7] | 4 (0.3%)  [0.1-0.7] | 0 (0%) | 0 (0%) | 2.0 (1.4)  1-3.6 |
|  | 4-month | 4 (0.3%)  [0.1-0.8] | 4 (0.3%)  [0.1-0.8] | 0 (0%) | 0 (0%) | 1.0 (0.3)  0.7-1.3 |
|  | 12-month | 3 (0.4%)  [0.1-1.0] | 3 (0.4%)  [0.1-1.0] | 0 (0%) | 0 (0%) | 1.1 (0.6)  0.7-1.5 |
|  | Median (IQR) study visits that drug is prescribed: 2 (2, 3) (inc. withdrawn residents), 2.5 (1.5, 3) (exc. withdrawn residents) | | | | | |
| Haloperidol | Baseline | 16 (1.1%)  [0.7-1.8] | 7 (0.5%)  [0.2-1.0] | 9 (0.6%)  [0.3-1.2] | 0 (0%) | 4.3 (4.2)  1-15 |
|  | 4-month | 16 (1.3%)  [0.8-2.1] | 9 (0.7%)  [0.4-1.4] | 7 (0.6%)  [0.3-1.2] | 0 (0%) | 6.0 (8.9)  1-30 |
|  | 12-month | 13 (1.5%)  [0.8-2.6] | 6 (0.7%)  [0.3-1.6] | 7 (0.8%)  [0.4-1.7] | 0 (0%) | 2.9 (3.3)  [1-11.5] |
|  | Median (IQR) study visits that drug is prescribed: 3 (1, 3) (inc. withdrawn residents), 3 (2, 3) (exc. withdrawn residents) | | | | | |
| Levomepromazine | Baseline | 4 (0.3%)  [0.1-0.7] | 0 (0%) | 4 (0.3%)  [0.1-0.7] | 0 (0%) | 137.5 (147.4)  12.5-300 |
|  | 4-month | 2 (0.2%)  [0.0-0.6] | 0 (0%) | 2 (0.2%)  [0.0-0.6] | 0 (0%) | 81.3 (97.2)  12.5-150 |
|  | 12-month | 3 (0.4%)  [0.1-1.0] | 1 (0.1%)  [0.0-0.6] | 2 (0.2%)  [0.0-0.8] | 0 (0%) | 25.0 (0.0) |
|  | Median (IQR) study visits that drug is prescribed: 3 (3, 3) (inc. withdrawn residents), 1 (1, 1) (exc. withdrawn residents) | | | | | |
| Olanzapine | Baseline | 23 (1.6%)  [1.1-2.4] | 23 (1.6%)  [1.1-2.4] | 0 (0%) | 0 (0%) | 6.7 (5.9)  2.5-20 |
|  | 4-month | 19 (1.6%)  [0.9-2.4] | 19 (1.6%)  [0.9-2.4] | 0 (0%) | 0 (0%) | 6.7 (6.3)  2.5-20 |
|  | 12-month | 17 (2.0%)  [1.2-3.2] | 17 (2.0%)  [1.2-3.2] | 0 (0%) | 0 (0%) | 6.9 (6.0)  2.5-20 |
|  | Median (IQR) study visits that drug is prescribed: 3 (2, 3) (inc. withdrawn residents), 3 (2, 3) (exc. withdrawn residents) | | | | | |
| Promazine | Baseline | 14 (1.0%)  [0.6-1.7] | 6 (0.4%)  [0.2-0.9] | 7 (0.5%)  [0.2-1.0] | 1 (0.1%)  [0.0-0.5] | 63.2 (47.9)  10-200 |
|  | 4-month | 9 (0.7%)  [0.4-1.4] | 5 (0.4%)  [0.2-1.0] | 2 (0.2%)  [0.0-0.7] | 2 (0.2%)  [0.0-0.7] | 53.9 (58.2)  10-200 |
|  | 12-month | 5 (0.6%)  [0.2-1.4] | 3 (0.4%)  [0.1-1.1] | 2 (0.2%)  [0.1-0.9] | 0 (0%) | 85.0 (69.8)  25-200 |
|  | Median (IQR) study visits that drug is prescribed: 2.5 (2, 3) (inc. withdrawn residents), 3 (2, 3) (exc. withdrawn residents) | | | | | |
| Quetiapine | Baseline | 58 (4.1%)  [3.2-5.2] | 55 (3.9%)  [3.0-5.0] | 1 (0.1%)  [0.0-0.5] | 2 (0.1%)  [0.0-0.6] | 69.6 (61.3)  10-300 |
|  | 4-month | 54 (4.4%)  [3.4-5.8] | 52 (4.3%)  [3.3-5.6] | 1 (0.1%)  [0.0-0.6] | 1 (0.1%)  [0.0-0.6] | 75.2 (76.2)  12.5-400 |
|  | 12-month | 39 (4.6%)  [3.3-6.2] | 38 (4.4%)  [3.2-6.0] | 0 (0%) | 1 (0.1%)  [0.0-0.6] | 68.9 (81.5)  12.5-400 |
|  | Median (IQR) study visits that drug is prescribed: 2 (2, 3) (inc. withdrawn residents), 3 (2, 3) (exc. withdrawn residents) | | | | | |
| Risperidone | Baseline | 106 (7.4%)  [6.2-8.9] | 103 (7.2%)  [6.0-8.7] | 3 (0.2%)  [0.1-0.7] | 0 (0%) | 0.9 (0.8)  0.25-6 |
|  | 4-month | 86 (7.1%)  [5.8-8.7] | 81 (6.7%)  [5.4-8.2] | 3 (0.2%)  [0.1-0.8] | 2 (0.2%)  [0.0-0.7] | 1.0 (0.9)  0.25-6 |
|  | 12-month | 65 (7.6%)  [6.0-9.6] | 57 (6.7%)  [5.2-8.5] | 4 (0.5%)  [0.2-1.2] | 4 (0.5%)  [0.2-1.2] | 1.2 (1.1)  0.25-6.25 |
|  | Median (IQR) study visits that drug is prescribed: 2 (1, 3) (inc. withdrawn residents), 3 (2, 3) (exc. withdrawn residents) | | | | | |
| Sulpiride | Baseline | 5 (0.4%)  [0.1-0.8] | 5 (0.4%)  [0.1-0.8] | 0 (0%) | 0 (0%) | 140 (54.8)  100-200 |
|  | 4-month | 5 (0.4%)  [0.2-1.0] | 5 (0.4%)  [0.2-1.0] | 0 (0%) | 0 (0%) | 108.0 (57.6)  40-200 |
|  | 12-month | 3 (0.4%)  [0.1-1.1] | 3 (0.4%)  [0.1-1.1] | 0 (0%) | 0 (0%) | 180.0 (192.9)  40-400 |
|  | Median (IQR) study visits that drug is prescribed: 2.5 (1, 3) (inc. withdrawn residents), 3 (2, 3) (exc. withdrawn residents) | | | | | |
| Trifluoperazine | Baseline | 2 (0.1%)  [0.0-0.6] | 2 (0.1%)  [0.0-0.6] | 0 (0%) | 0 (0%) | 6.5 (4.9)  3-10 |
|  | 4-month | 2 (0.2%)  [0.0-0.7] | 2 (0.2%)  [0.0-0.7] | 0 (0%) | 0 (0%) | 6.5 (4.9)  3-10 |
|  | 12-month | 1 (0.1%)  [0.0-0.6] | 1 (0.1%)  [0.0-0.6] | 0 (0%) | 0 (0%) | 5.0 (0.0) |
|  | Median (IQR) study visits that drug is prescribed: 2.5 (2, 3) (inc. withdrawn residents), 3 (3, 3) (exc. withdrawn residents) | | | | | |

Supplementary table 4. Number of prescriptions at baseline for PRN psychotropics that recorded an indication suggestive of agitation/distress/anxiety, and other or missing indications

| Drug | Drug class | Indication on medication chart (n) | | |
| --- | --- | --- | --- | --- |
|  |  | Agitation/ distress/ anxiety | Other | Missing |
| **Antidepressants (%)** | **n=2** | **100.0** | **0.0** | **0.0** |
| Trazodone (n) | Antidepressant | 2 | 0 | 0 |
| **Antipsychotics (%)** | **n=27** | **25.9** | **0.0** | **74.1** |
| Haloperidol | Antipsychotic | 1 | 0 | 8 |
| Levomepromazine | Antipsychotic | 0 | 0 | 4 |
| Promazine | Antipsychotic | 4 | 0 | 4 |
| Quetiapine | Antipsychotic | 1 | 0 | 2 |
| Risperidone | Antipsychotic | 1 | 0 | 2 |
| **Anxiolytics/hypnotics (%)** | **n=162** | **37.7** | **8.6** | **53.7** |
| Clobazam | Benzodiazepine | 0 | 0 | 1 |
| Clonazepam | Benzodiazepine | 1 | 0 | 3 |
| Diazepam | Benzodiazepine | 13 | 1^3^ | 15 |
| Lorazepam | Benzodiazepine | 38 | 5^2^ | 39 |
| Midazolam | Benzodiazepine | 8 | 2^1^ | 12 |
| Oxazepam | Benzodiazepine | 1 | 0 | 3 |
| Temazepam | Benzodiazepine | 0 | 0 | 1 |
| Zopiclone | Hypnotic | 0 | 6^4^ | 13 |

^1^Pain (n=1), comfort/symptom control (n=1) ^2^Pain (n=2), end of life (n=2), mood stabilizer (n=1) ^3^Pain management (n=1) ^4^Sleep aid
